# Symptom burden and health-related quality of life six months after hyperbaric oxygen therapy in cancer survivors with pelvic radiation injuries

**DOI:** 10.1007/s00520-022-06994-8

**Published:** 2022-03-23

**Authors:** Grete K. Velure, Bernd Müller, May Aa. Hauken

**Affiliations:** 1 Centre for Crisis Psychology, Faculty of Psychology, University of Bergen, Møllendalsbakken 9, N - 5009 Bergen, Norway; 2 Hyperbaric Medicine Unit, Department of Occupational Medicine, Haukeland University Hospital, Bergen, Norway

**Keywords:** Hyperbaric oxygen treatment, Pelvic malignancies, Pelvic radiotherapy, Quality of life, Side effects

## Abstract

**Purpose:**

Late radiation tissue injuries (LRTIs) after treatment for pelvic cancer may impair health related quality of life (HRQoL). Hyperbaric oxygen therapy is an adjuvant therapy for LRTIs, but limited studied. The aim of this study was to explore the development and association between symptoms of LRTI and HRQoL following hyperbaric oxygen treatment.

**Methods:**

A pretest–posttest design was used to evaluate the changes in pelvic LRTIs and HRQoL from baseline (T1), immediately after treatment (T2) and at six-month follow-up (T3). EPIC and EORTC-QLQ-C30 were used to assess LRTIs and HRQoL. Changes were analysed with *t*-tests, and associations with Pearson’s correlation and multiple regression analyses.

**Results:**

Ninety-five participants (mean age 65 years, 52.6% men) were included. Scores for urinary and bowel symptoms, overall HRQoL, all function scales and the symptoms scales sleep, diarrhoea, pain and fatigue were significantly improved six months after treatment (*P*-range = 0.00–0.04). Changes were present already at T2 and maintained or further improved to T3. Only a weak significant correlation between changes in symptoms and overall HRQoL was found (Pearson *r-*range 0.20–0.27).

**Conclusion:**

The results indicate improvement of pelvic LRTIs and HRQoL following hyperbaric oxygen therapy, corresponding to minimal or moderate important changes. Cancer survivors with pelvic LRTIs and impaired HRQoL may benefit from undergoing hyperbaric oxygen therapy. Especially the reduced symptom-severity and improved social- and role function can influence daily living positively.

**Trial registration:**

ClinicalTrials.gov: NCT03570229. Released 2. May 2018.

## Introduction

Radiotherapy is an important part of the multimodal curative treatment for pelvic cancers (e.g. urological, bowel and gynaecological cancers), but late radiation tissue injuries (LRTIs) may develop months or years later [1–3]. This includes cystitis, proctitis/enteritis, soft tissue necrosis, osteoporosis and fistulas, with symptoms including increased frequency, urgency and leakage of urine and faeces, diarrhoea, haematuria, osteoradionecrosis and pain [4, 5]. Treatment options for LRTIs are limited and consist mainly of prophylactic measures and symptomatic treatment (e.g. local or systemic pharmacological, surgical, physiotherapeutic training and behavioural adaptions) [6, 7]. However, hyperbaric oxygen therapy has shown positive effects on a range of LRTIs, including soft tissue necrosis, cystitis and proctitis, based on its ability to increase tissue oxygenation, stimulate angiogenesis and cellular regeneration and thereby induce revitalising and healing of damaged tissue [8–10].

As late effects from cancer treatment may affect all parts of cancer survivors’ life, HRQoL has emerged as an important indicator of healthcare outcomes [11]. HRQoL is commonly defined as an individual, subjective, multidimensional, dynamic and interrelated concept consisting of physiological, psychological and social aspects of well-being [12, 13]. Distress from LRTI symptoms is therefore likely to influence the different dimensions and overall HRQoL negatively, while improvements in symptoms or any other dimension may promote HRQoL positively. Previous studies, including research from our group, show that pelvic LRTIs across cancer types impair cancer survivors HRQoL, with higher radiation-toxicity, combinations of chemotherapy and radiotherapy and higher symptom burden as risk factors [2, 14, 15]. Especially gastrointestinal symptoms seem to severely impair HRQoL [16]. Although some studies have demonstrated positive associations between symptom improvement and HRQoL after hyperbaric oxygen therapy, results are conflicting and more research is needed [8, 17, 18]. The goal of this study was to explore the development of symptom severity and HRQoL following hyperbaric oxygen therapy, as well as the associations between these over times.

## Methods

### Study design, recruitment and eligibility criteria

This study is part of a prospective longitudinal study with an overarching aim to increase the understanding of pelvic LRTIs in cancer survivors undergoing hyperbaric oxygen therapy (trial registration: ClinicalTrials.gov. NCT03570229). In the study at hand, we used a pretest–posttest design in order to assess changes in the development of pelvic LRTI symptoms and HRQoL after hyperbaric oxygen therapy.

The study sample was recruited from all cancer survivors with pelvic LRTIs (proctitis, cystitis, osteoradionecrosis, wounds and fistulas) assigned to hyperbaric oxygen therapy at The Norwegian National Unit for Planned Hyperbaric Oxygen Therapy between August 2018 and March 2021. Inclusion criteria were as follows: (a) pelvic radiation injury after intended curative radiation for pelvic cancer (prostate, gynaecological, urological, bowel and bone cancers); (b) LRTI symptoms from bowel, bladder or pelvic area, with signs of radiation injury verified by endoscopy or radiology; (c) ≥ 6 months from finished radiation; (d) aged ≥ 18 years. Exclusion criteria were as follows: (a) severe physical and/or mental comorbidity representing a contraindication for hyperbaric oxygen therapy including signs of active cancer; (b) insufficient language skills to complete study questionnaires; (c) previously treated with hyperbaric oxygen.

### Hyperbaric oxygen therapy

During hyperbaric oxygen therapy, patients are placed in a pressure chamber and breathe 100% oxygen while exposed to elevated ambient pressure [9]. The side effects of hyperbaric oxygen therapy are usually minimal and temporary, limited mostly to mild middle ear barotrauma and transient visual disturbance [19]. In the present study, the participants received hyperbaric oxygen therapy in a mono-place chamber, breathing pure oxygen at a pressure of 2.4 atmosphere absolute for 90 min once a day (Monday–Friday) for six weeks.

### Data collection

Data were collected by self-report questionnaires at baseline (T1), at the end of the six-week hyperbaric oxygen therapy course (T2), and at follow-up six months after treatment (T3).

Pelvic LRTI symptoms were measured with the Expanded Prostate Cancer Index Composite (EPIC), urinary and bowel domain [20]. This is a self-report questionnaire on urinary and bowel symptoms based on the past four weeks [21, 22]. Items are scored on Likert scales, with different response categories (0–4, 1–3, 1–4 and 1–5), and transformed to a 0–100 score [23]. A total score for each domain, as well as urinary sub scores (function, bother, incontinence, irritation/obstruction) and bowel subscales (function, bother), is calculated by the mean of all included items. A lower score indicates more severe symptoms. The instrument has shown to be valid, reliable and sensitive to assess urinary and bowel toxicity and complications from radiotherapy for prostate cancer and gynaecological malignancies (Cronbach’s alpha range between 0.82 and 0.86) [20, 21]. The minimal clinically important changes in the EPIC urinary domain are stated to be between 6 and 9 points and between 4 and 6 points in the bowel domain [24]. In healthy controls, mean urinary scores of 89.5 (SD 11.2) and bowel scores of 92.5 (SD 8.7) have been reported [25].

HRQoL was measured using the European Organization for Research and Treatment of Cancer (EORTC) quality of life questionnaire (QLQ-C30, version 3.0) [26]. This is a self-report questionnaire consisting of 30 questions. Items are scored on Likert scales with different response categories (1–4, 1–7) and transformed into a 0–100 score. The scores are combined into five functional scales (physical, role, emotional, cognitive and social), nine symptom scales (fatigue, pain, nausea/vomiting, dyspnoea, sleep, appetite loss, constipation, diarrhoea and financial difficulties) and one overall HRQoL scale [27]. A high score reflects a high level of function or overall HRQoL, while high scores on the symptom scales represent a high symptom burden associated with poor HRQoL. This instrument is widely used with documented robust psychometric properties (Cronbach’s alpha range between 0.80 and 0.90 for most multi-item scales and single items) [28]. Changes are categorised as minimal clinically important if 5–10 points, moderate if 10–20 points and very much if > 20 points [27, 29]. Overall HRQoL scores of the general population have been reported to be at mean 71.2 (SD 22.4) [28].

To ensure an acceptable study participation burden and that the questions were comprehendible and perceived relevant, the questionnaires were tested with four cancer survivors with pelvic LRTIs who were previously treated with hyperbaric oxygen and not participating in the study. They provided positive feedback on the relevance, content, comprehensibility and length of the questionnaires, and did not offer any suggestions for improvements.

### Statistics

Descriptive continuous data are presented as means and categorical data as frequencies. All variables were normally distributed and determined by histograms and skewness. Cronbach’s alpha was high for both instruments (*α* = 0.80–0.89). The few missing data were not replaced.

Differences in pelvic LRTI symptoms and HRQoL between the time points T1, T2 and T3 were analysed by paired-samples *t*-test [30]. As a value of less than 80 points in the urinary and bowel domain of the EPIC indicates a significant symptom burden, separate analyses were performed for the respective subgroups (EPIC < 80 at T1) [31]. Development over time is presented as mean change of scores, with 95% confidence intervals.

To assess the correlation of the development in pelvic LRTI symptoms with overall HRQoL, Pearson’s correlation analysis was used [32]. Multiple linear regression analysis was carried out to explore the relationship between changes in overall HRQoL as dependent variable and changes in pelvic LRTI symptoms as independent variables. *P*-values ≤ 0.05 indicate statistically significant findings. All analyses were performed using IBM SPSS Statistics for Windows version 26 [33].

### Ethical considerations

The study was approved by the Regional Committee for Medical and Health Research Ethics, (Nothern Norway) (2018/706) and was conducted in compliance with the Declaration of Helsinki and the requirement for data processing and handling [34]. The participants received written information about the study, and all gave written consent.

## Results

### Sample characteristics

In total, 125 participants met the eligibility criteria, and 95 consented to participate in the study. Non-participation was related to decline (*n* = 11), withdrawal from treatment (*n* = 8), previous hyperbaric oxygen therapy (*n* = 5) and loss to follow-up (*n* = 6; one died, two did not return questionnaires and three discontinued treatment due to other illness). Sample characteristics are outlined in Table 1. All participants completed the six-week hyperbaric oxygen therapy course.

**Table 1:** Sample characteristics (*N* = 95)

	*n* (%)
Gender	
Male	50 (52.6)
Female	45 (47.4)
Age, years (mean (SD, range))	65 (11.6, 32–84)
Work status	
Sick leave/disability pension/retired	78 (82.1)
Full time/part time employment	17 (17.9)
Civil status	
Married/cohabiting	67 (70.5)
Single	28 (29.5)
Children under 18 years of age	
No	84 (88.4)
Yes	11 (11.6)
Medical characteristics	
Cancer site	
Prostate	49 (51.6)
Gynaecological	34 (36.0)
Rectum/anus	12 (12.4)
Referral diagnosis	
Cystitis and proctitis	54 (56.84)
Proctitis	25 (26.32)
Cystitis	11 (11.58)
Osteoradionecrosis pelvis	5 (5.26)
Type of cancer treatment	
Radiation only	61 (64.2)
Chemotherapy and radiation	34 (35.8)
Types of radiation	
External only	66 (69.5)
External and internal	29 (30.5)
Radiation dose, Gy (range)	
External	35.0–100.0
Internal	7.0–75.0
Months since radiation (median (range))	47.0 (7–511)

Abbreviations: *Gy*, Gray; *n*, total number of participants; *n*, number of participants; *SD*, standard deviation

### Pelvic LRTI symptoms

LRTI symptom scores during the study period are presented in Table 2.

**Table 2:** Urinary and bowel symptom scores and health-related quality of life scores at baseline and after hyperbaric oxygen therapy (*N* = 95)

HRQoL		Mean (SD) values			Mean (SD) values norm populations	Mean change from T1 to T3 (95% CI) *P*	
EPIC total urinary/bowel ^a^		Baseline (T1)	Six weeks (T2)	Six months (T3)	Controls without cancer^c^	T1–T3	*P*
Urinary		70.0 (17.2)	72.9 (18.5)**	75.3 (17.3)	89.5 (11.2)	5.3 (2.3; 8.3)	< 0.00
Bowel		63.4 (13.4)	67.4 (14.7)**	70.0 (16.6)	95.5 (9.5)	6.7 (3.7; 9.6)	< 0.00
Urinary score < 80 at T1 (*n* = 65)		60.4 (11.8)	65.3 (17.0)***	70.5 (17.8)***		10.1 (6.4; 13.7)	< 0.00
Bowel score < 80 at T1 (*n* = 79)		60.1 (10.9)	64.9 (13.6)***	67.5 (16.0)		7.4 (4.1; 10.7)	< 0.00
EORTC QLQ-C30^b^					General population^d^		
Overall HRQoL		54.7 (21.7)	61.3 (19.9)^**^	61.8 (20.0)	71.2 (22.4)	7.1 (2.5; 11.7)	< 0.00
Function	Physical	69.3 (23.7)	71.9 (24.2)	72.8 (24.2)	89.8 (16.2)	3.5 (0.3; 6.7)	0.03
	Role	60.8 (35.1)	65.9 (28.7)*	67.2 (28.4)	84.7 (25.4)	6.4 (0.4; 12.4)	0.04
	Emotional	73.3 (24.6)	81.1 (21.3)***	77.4 (23.5)*	76.3 (22.8)	4.1 (0.1; 8.2)	0.04
	Cognitive	73.3 (27.0)	75.4 (23.7)	78.1 (23.6)	86.1 (20.0)	4.8 (0.5; 9.2)	0.02
	Social	48.8 (31.8)	62.2 (32.2)***	63.4 (31.7)	87.5 (22.9)	14.6 (8.4; 20.6)	< 0.00
Symptoms	Fatigue	49.1 (28.4)	46.8 (26.9)	40.1 (27.1)***	24.1 (24.0)	− 9.0 (− 4.1; − 13.9)	< 0.00
	Pain	40.3 (32.2)	31.6 (29.7)**	31.1 (29.8)	20.9 (27.6)	− 9.2 (− 3.6; − 14.9)	< 0.00
	Nausea/vomiting	9.4 (16.1)	5.4 (9.65)**	5.1 (11.3)	3.7 (11.7)	− 4.3 (− 1.1; − 7.5)	< 0.00
	Dyspnoea	28.1 (29.1)	27.4 (27.6)	22.2 (27.4)*	11.8 (22.8)	− 5.9 (− 0.6; − 11.2)	0.03
	Sleep disturbance	49.3 (3.3)	39.5 (32.0)***	36.2 (32.7)	21.8 (29.7)	− 13.1 (− 6.8; − 19.3)	< 0.00
	Appetite loss	13.7(21.6)	13.4 (23.2)	10.1 (20.8)	6.7 (18.3)	− 3.6 (− 0.9; − 8.1)	0.11
	Constipation	27.7 (32.1)	29.2 (44.4)	24.1 (28.7)	6.7 (18.4)	− 3.6 (− 10.4; 3.0)	0.27
	Diarrhoea	52.7 (35.3)	38.3 (31.8)***	40.9 (34.2)	7.0 (18.0)	− 11.8 (− 5.2; − 18.3)	< 0.00
	Financial impact	19.3 (32.6)	14.7 (30.3)*	16.9 (32.8)	9.5 (23.3)	− 2.6 (− 7.6; 2.4)	0.31

Mean (SD) values derived from descriptive statistics. Mean change scores over time (95% CI, *P*) derived from *t*-tests

Abbreviations: *Bowel score* < *80*, scoring less than 80 points at baseline in the bowel domain of the Expanded Prostate Index Composite; *CI*, confidence intervals; *EORTC QLQ-C30*, European Organization for Research and Treatment of Cancer Quality of Life Questionnaire; *EPIC*, the Expanded Prostate Index Composite (scores 0–100); *HRQoL*, health-related quality of life (scores 0–100); *P*, the mean difference is significant at the 0.05 level; *SD*, standard deviation; *Urinary score* < *80*, scoring less than 80 points at baseline in the urinary domain of the Expanded Prostate Index Composite

^a^EPIC, minimal clinically important change; urinary total, range 6–9 points; bowel total, range 4–6 points [24]

^b^EORTC QLQ-C30, minimal clinically important change, range 5–10 points; moderate change, range 10–20 points; very much change, range > 20 points [28]

^c^EPIC, control population [25]

^d^EORTC-QLQ C30, reference values manual [28]

^*^p < 0.05; **p < 0.01; *** *p* < 0.001 for significance level of differences between T1 and T2 and between T2 and T3

At baseline, mean urinary and bowel total scores were clearly below the threshold generally regarded as significant symptomatic [31]. At six-month follow-up, urinary and bowel symptom scores had increased with 5.3 and 6.7 points, respectively, which is within the range of minimal clinically important changes.

Participants scoring less than 80 points in the EPIC urinary or bowel domain at baseline scored approximately 20–30 points below this threshold, indicating the more severe symptom burden. In these groups, the urinary and bowel symptoms improved with 10.1 and 7.4 points, respectively, an improvement above the minimal clinically important change. A statistically significant, but less pronounced improvement of urinary and bowel symptoms was found already at T2, at the end of hyperbaric oxygen therapy.

### HRQoL

The changes in HRQoL scores are presented in Table 2. The participants reported severely impaired overall HRQoL at baseline compared to the general population (mean 54.7 vs. 71.2). Overall HRQoL scores increased with 7.1 points from baseline to six-month follow-up corresponding to a minimal clinically important change. Interestingly, this increase was present already at the end of the hyperbaric oxygen therapy period.

At baseline, all function scale scores were below the scores of the general population, which indicates clinically important impairments. All these scores improved significantly at six-month follow-up. The increase in social function of 14.5 points corresponds to a moderate change, and the increase in role function of 6.4 points corresponds to a minimal clinically important change.

All HRQoL symptom scale scores at baseline were above the scores of the general population, indicating more symptoms, but most scores improved significantly after treatment. At six-month follow-up, scores for sleep disturbance and diarrhoea had decreased with − 13.1 and − 11.8, respectively, which corresponds to a moderate change. Pain and fatigue scores decreased with − 9.3 and − 9.0, which corresponds to a minimal clinically important change.

The largest improvements within all functional dimensions and most symptom scales were observed at the end of the treatment and were maintained at six-month follow-up. Emotional function scores decreased between the end of the treatment to six-month follow-up at T3. However, the scores at T3 were still statistically significantly higher than the baseline scores; i.e. an improvement was maintained.

### Associations between changes in LRTI symptoms and overall HRQoL

Associations between changes in LRTI symptoms and overall HRQOL are presented in Table 3.

**Table 3:** Multiple regression analysis of changes in EPIC urinary and bowel symptom scores and overall HRQoL from baseline (T1) to six-month follow-up (T3)

Change Overall HRQoL^a^ from T1 to T3	Pearson *r* (*P*)	B (SE B)	*β* (*P*)	Multicollinearity	*r*^2^
*Multiple regression*					0.10
Change EPIC urinary total from T1 to T3	0.27 (0.00)	0.37 (0.2)	0.22 (0.07)	0.83	
Change EPIC bowel total from T1 to T3	0.20 (0.04)	0.17 (0.19)	0.11 (0.37)	0.83	
*Correlation only:*					
Change EPIC urinary score < 80 at BL from T1 to T3	0.24 (0.03)				
Change EPIC bowel score < 80 at BL from T1 to T3	0.23 (0.02)				

Abbreviations: *B*, unstandardised regression coefficient; *BL*, baseline; *EPIC*, Expanded Prostate Cancer Index Composite; *HRQoL*, health-related quality of life; *Multicollinearity*, tolerance factor; *P*, significance level; *r*^*2*^, explained variance; *SE B*, standard error of the coefficient; *β*, standardised coefficient

^a^Dependent variable

Overall, the observed changes in urinary and bowel symptoms and overall HRQoL showed a significant, but weak positive correlation. In the multiple linear regression analysis, changes in urinary and bowel symptoms from baseline (T1) to six-month (T3) follow-up explained only 10% of the variance of overall HRQoL.

## Discussion

The development of symptom severity and HRQoL following hyperbaric oxygen therapy and the associations between these have been subject to limited research. This study adds to this knowledge base, and the main findings are discussed below.

At a median time of nearly four years following radiotherapy, the participants reported severe urinary and bowel symptoms compared to healthy controls [25]. Six months after hyperbaric oxygen therapy, symptom severity was significantly improved. Similar results have been shown in previous studies [8, 17], although the changes shown in the present study were less pronounced than the changes found in the RICH-ART study by Oscarsson et.al [8]. This may be explained by the fact that the RICH-ART study included cancer survivors with radiation cystitis with more pronounced urinary symptoms, while our study included individuals with a broader range of pelvic LRTI symptoms and consequently less severe urinary symptoms. Despite relatively small changes in urinary and bowel symptoms in our sample, the changes were noticeable (both clinically and statistically significant) to the participants. Previous research has shown that patients with pelvic LRTIs appreciate and welcome any improvements in symptom severity, even if small [35].

An interesting finding was that more than half of the participants (56.8%) scored less than 80 points on both urinary and bowel symptoms at baseline. This supports the notion that pelvic LRTIs may be part of a pelvic syndrome with simultaneous affection of multiple organs. Not unexpectedly, participants with the most severe symptoms (urinary or bowel < 80) at baseline reported a larger symptom improvement after treatment. This aligns to previous research [36], and may thus give an indication of which patients might benefit the most and should be referred to hyperbaric oxygen therapy. These findings are also important for healthcare professionals with respect to patient information and clarifying expectations of hyperbaric oxygen therapy.

At baseline, the participants reported severely impaired HRQoL parameters compared to the general population [28], suggesting that their daily life was highly compromised and that supporting interventions seemed needed. Six months after hyperbaric oxygen therapy, overall HRQoL, all function scales and most symptom scores were significantly improved and closer to those of the general population [28]. A noticeable (both clinically and statistically significant) improvement was particularly observed for social- and role function, which deals with severity and interference in daily life, for example related to being out of work, social activities and/or family life and household tasks [37]. It is likely that decreased symptom severity such as less diarrhoea, urge, pain and improved sleep quality increase the survivors’ ability to be social active and increase their role participation. This is supported in literature stating that improvement in bodily and structural dimensions facilitates improvement in activity and participation [38]. However, despite a clinically significantly improvement in pain and diarrhoea, the scores were still high and clearly above the general population at six-month follow-up [28], underlining that the participants still experienced noticeable symptom burden.

Socioeconomic factors such as unemployment are important social determinants in health, where research particularly indicates a relationship between urinary incontinence and work status [39]. In our study, only a minority of the participants worked part- or full time. This can partly be explained by the participants’ age and retirement. However, most participants in working age were on sick leave or disability pension. Research shows that physical late effects and fatigue after cancer treatment continue to impair work ability among cancer survivors, affect career and increase economical stress [40]. Consequently, improvement in symptom severity, social- and role function seem to be important factors in considerations of return to work and work ability.

In addition, the findings revealed that emotional function improved significantly at the end of hyperbaric treatment. Already during the therapy course, the participants experienced improvement in symptoms (e.g. diarrhoea and pain) which may have contributed to reduced emotional distress. Furthermore, they met other patients in the same situation or with even more severe symptoms. Being in an environment with peers may itself have influenced emotional function positively, due to sharing of common experiences, socialising and supporting each other. Cancer survivors often feel left alone with their late effects and peer support has shown to be important for promoting positive changes, improving psychosocial function, empowerment and HRQoL [41–43]. Daily professional follow-up for several weeks during the therapy course may also have had a positive impact on the participants’ emotional function [42]. The professionals’ expertise, offering a combination of knowledge and opportunities for asking questions to medical professionals, is an essential factor in promoting emotional functioning and making patients feel comfortable and safe [35, 41]. These notions seem to be supported by the fact that emotional function scores decreased from the end of the treatment to six-month follow-up. It seems that returning to daily life after the treatment may have increased a feeling of being left alone and perhaps also increased emotional stress regarding remaining symptoms and concerns of daily life [44, 45]. This highlights the importance of support in coping with emotional issues, which has a documented impact on both physical and psychological well-being in cancer survivorship [38, 42].

It is interesting that the largest improvements also for overall HRQoL, all functional scales and most symptom scales were observed already at the end of hyperbaric oxygen therapy. This could have several explanations. First, experiences of symptom relief during treatment and the fact that the participants had just completed a six-week treatment course may have given hope for a more normalised everyday life. Hope and anticipations are known to play a predominant role in HRQoL [12, 46]. Second, getting a specific and causal explanation of their symptoms may have felt relieving, and research has shown that knowledge is an essential factor in coping [47, 48]. These factors, in addition to peer support and the professional follow-up, as mentioned above, may also be plausible in contributing to increased HRQoL immediately after treatment. In sum, the circumstances around the treatment with hyperbaric oxygen, the medical care, social aspects and close professional follow-up may all have contributed to improvement of HRQoL as well as the treatment itself [35].

The changes in symptom severity were significantly positively associated with changes in overall HRQoL, but the correlation was surprisingly weak. It may be questioned if the symptom burden has as much direct influence on overall HRQoL as previously expected. Here, both the amount of improvement and the severity of the remaining symptoms may be relevant. Furthermore, overall HRQoL had improved the most already at the end of the treatment, while the urinary and bowel symptoms improved through the whole course of the study. However, the overall improvement of HRQoL was maintained at six-month follow-up. An interesting question is therefore whether the improvement in overall HRQoL would have still been maintained at six-month follow-up if the symptoms had not improved, although no conclusion can be drawn here.

### Clinical implications

The results demonstrate a high symptom severity and impaired HRQoL before hyperbaric oxygen therapy and an improvement after the treatment. This may have several implications for cancer survivors, clinical practice and further research. Systematic assessment of pelvic LRTI symptoms and HRQoL after radiation should be part of routine follow-up, whereby impairments should be addressed with appropriate symptom management and supporting interventions. Second, the clinically significant improvement in symptoms and HRQoL parameters after hyperbaric oxygen therapy indicate that this treatment can be relevant for cancer survivors with pelvic LRTIs. In particular decreased symptom severity and improvement in social and role function can influence survivors’ day-to-day functioning positively. This is important knowledge for healthcare professionals, and may provide a basis for realistic information to patients, with the study suggesting that those with the most severe symptoms may benefit the most from hyperbaric oxygen therapy. Third, the improvement in HRQoL during the therapy course emphasises the importance of follow-up of cancer survivors in addition to appropriate pelvic LRTI symptom management. The benefits of meeting fellow patients, exchanging experiences and supporting each other seem to be important factors during the treatment course. Consequently, organising the hyperbaric oxygen therapy course in a way that enables peer support is of importance. Furthermore, the findings may also indicate that healthcare professionals’ support and follow-up promoted HRQoL positively.

In general, there is limited evidence on the use of hyperbaric oxygen therapy in survivors of pelvic cancer with LRTI, and more research in this field is highly needed. Measurements over a longer period of time would be useful to gain increased knowledge about long-term changes in symptoms and HRQoL after hyperbaric oxygen therapy. Additionally, mixed methods studies would be valuable in adding to the knowledge base within this field of research. The combination of quantitative data examine outcome variables with qualitative data exploring participants’ experiences would offer greater insight into the use of hyperbaric oxygen therapy in this group of patients.

### Limitations and strengths

The focus on a selected population referred to hyperbaric oxygen therapy limits the generalisation of the findings, and the pretest–posttest design did not allow for assessment of causal relationships. However, the instruments used to evaluate symptom burden and HRQoL are well recognised, and the high survey completion rates also strengthen the study. The study revealed clinical important and potential explanatory variables for improved symptom severity and HRQoL parameters.

## Conclusion

The results from this study indicate a beneficial outcome after hyperbaric oxygen therapy in patients with pelvic LRTIs concerning both pelvic symptoms and HRQoL. The observed changes were of small magnitudes but correspond to clinically significant improvements in both urinary and bowel symptoms after six months, with a noticeable improvement already at the end of hyperbaric treatment. The participants also reported an early positive influence on HRQoL after the treatment that was maintained six months later. Especially overall HRQoL, social- and role function, sleep disturbance, diarrhoea, pain and fatigue were improved after hyperbaric oxygen therapy, which is likely to lead to improvement in the daily life of the affected individuals. Changes in pelvic LRTIs were to a relatively small degree associated with changes in HRQoL.

## Data Availability

De-identified data will be available from the study leader on reasonable request after the end of the project.

## Declarations

### Clinical trial registration

ClinicalTrials.gov Identifier: NCT03570229. Released 2. May 2018.

### Standards of reporting

The researchers followed established guidelines in preserving anonymity and safe handling of the data as well as STROBE guidelines for reporting.

## Abbreviations

EORTC: European Organization for Research and Treatment of Cancer EPICthe Expanded Prostate Cancer Index Composite
HRQoL: Health-related quality of life
LRTIs: Late radiation tissue injuries
